# Wavelet-Based Color Pathological Image Watermark through Dynamically Adjusting the Embedding Intensity

**DOI:** 10.1155/2012/406349

**Published:** 2012-12-02

**Authors:** Guoyan Liu, Hongjun Liu, Abdurahman Kadir

**Affiliations:** ^1^Department of Dermatology, Affiliated Hospital of Weifang Medical University, Weifang 261031, China; ^2^Department of Information Engineering, Weifang Vocational College, Weifang 261041, China; ^3^School of Computer Science and Engineering, Xinjiang University of Finance and Economics, Urumqi 830012, China

## Abstract

This paper proposes a new dynamic and robust blind watermarking scheme for color pathological image based on discrete wavelet transform (DWT). The binary watermark image is preprocessed before embedding; firstly it is scrambled by Arnold cat map and then encrypted by pseudorandom sequence generated by robust chaotic map. The host image is divided into *n* × *n* blocks, and the encrypted watermark is embedded into the higher frequency domain of blue component. The mean and variance of the subbands are calculated, to dynamically modify the wavelet coefficient of a block according to the embedded 0 or 1, so as to generate the detection threshold. We research the relationship between embedding intensity and threshold and give the effective range of the threshold to extract the watermark. Experimental results show that the scheme can resist against common distortions, especially getting advantage over JPEG compression, additive noise, brightening, rotation, and cropping.

## 1. Introduction

For content owners and distributors, there emerged a necessary concern in regard to the content authentication of pathological images as well as copyright protection. A latent solution to this issue is bestowed by digital watermarking. In general, image watermarking can be divided into two categories, according to the processing domain of cover images where the watermark is embedded: (1) the spatial domain method, which directly modifies the intensity value of the image and these algorithms are simple and speedy but not robust; (2) the frequency domain method, which is to modify the frequency coefficients.

In recent years, chaos is employed to encrypt the image, for it has sensitive dependence on initial conditions and can be employed to generate pseudorandom sequences, so the algorithm has large key space. Shyamsunder and Kaliyaperumal [1] proposed an image encryption scheme, which incorporates the concept of modular arithmetic and chaos theory. A necessary random matrix is generated for image encryption, and the look-up table is used to find the element by modular inverse of the random matrix for decryption. Rawat and Raman [2] proposed a chaos-based watermarking scheme for image authentication and tamper detection. Their scheme can detect any modification made to the image and can also indicate the specific locations that have been modified. To improve the security of the proposed scheme, two chaotic maps are employed. Li et al. [3] used logistic system and Chebyshev maps to construct a hybrid chaotic mapping system, the aim is to set risk transfer, process and improve risk management efficiency in projects management. That is a good case for applying chaos in enterprise management. 

The DWT approach remains one of the most effective ways for image watermarking. Lin et al. [4] proposed a blind watermarking algorithm based on maximum wavelet coefficient quantization; the blocks are randomly selected from different subbands, by adding different energies to the maximum wavelet coefficient to embed the watermark. The wavelet functions will analyze image features such as edges and borders through good space-frequency localization. They are used in several fields: image compression, signal denoising, image smoothing, and texture analysis. The main advantages of embedding watermarks in the DWT can be found in [5, 6].

The most important issue in DWT-based image watermarking is how to choose the effective coefficients to be embedded and extracted. Liu [7] estimates the noise detection threshold of each wavelet coefficient in luminance and chrominance components of color image. The thresholds are derived into a locally dynamic fashion based on the wavelet decomposition, through which the perceptually significant coefficients are selected for embedding watermark. Al-Otum and Samara [8] proposed a watermark scheme based on the wavelet trees, which exploits the significant features and relations between the color pixel components in the wavelet domain. The watermark is embedded by spreading it that the interpixel robust relations carry the watermark bit sign with sufficient energy. Zhang et al. [9] proposed an adaptive block-based for embedding binary watermark into grayscale image. In these articles, the watermark bits are only shuffled by pseudorandom sequence, they are not scrambled to uniformly, and randomly distributed in the host image, which will lead to be attacked easily. The threshold is a fixed experimental value, and the authors did not explain how to get the threshold.

Horng et al. [10] proposed a blind watermarking algorithm based on the significant difference of wavelet coefficient quantization. The maximum wavelet coefficients are quantized that their significant difference between watermark bit 0 and 1 exhibits a large energy difference, which can be used for watermark extraction. An adaptive threshold is designed to extract the watermark.

 In this paper, we propose a dynamic blind watermarking scheme for color pathological image based on DWT; the watermark is scrambled firstly by Arnold cat map and XOR with pseudorandom sequence generated by Chebyshev map. The host image is divided into *n* × *n* blocks, and each bit of the encrypted watermark is embedded into the detail wavelet sub-band, that is, the higher-frequency domain of blue component. We modify the wavelet coefficient dynamically according to the mean and variance of the subbands to embed “0” or “1.” In addition, we research the relationship between the embedding intensity and the threshold and deduce the range of the threshold, by which to extract the watermark correctly.

## 2. Watermarking Algorithm

### 2.1. Preprocessing the Watermark Image

 The watermark used for embedding is a binary logo image, which is very small in size compared to the host image. The watermark needs to be very small so that it is spatially localized and becomes robust against the intentional and unintentional attacks.

For a binary logo image, in order to uniformly and randomly spread the bits over the host image, it is needed to be preprocessed, or the watermarked image cannot resist against even the simplest attack: cropping. The watermark can be preprocessed in numerous ways, such as randomly select the embedding position, generate pseudorandom sequences to shuffle these bits [11], or the original watermark itself is dynamically generated from a pseudorandom Gaussian sequence [12, 13].

Here we take two measures to preprocess the watermark bits. Firstly, in order to make the watermarked image be more robust to resist against cropping attack, the two-dimensional Arnold cat map [14] is employed to scramble these binary bits. Suppose the watermark is an *n* × *n* binary image *W*; after scrambling, we get the binary image *W*_*A*_; the aim is to uniformly spread the watermark bits in the host image. By this means, the watermarked image can be more robust against cropping than the method in [7]. The iteration times *t* is served as one of the keys. The Arnold cat map can be described as follows:

(1) $$\begin{array}{c} \left[\begin{array}{c} x_{n+1}\\ y_{n+1} \end{array}\right]=\left[\begin{array}{cc} 1 & 1\\ 1 & 2 \end{array}\right]\left[\begin{array}{c} x_{n}\\ y_{n} \end{array}\right]\mathrm{mod}n. \end{array}$$

Secondly, watermarks generated from low-pass chaotic signals have superior performance over other signal types [13]. In order to eliminate the statistical significance and make the numbers of 0 and 1 approximately equal, a pseudorandom sequence *S*_*n*_ ∈ {0,1} will be generated by the Chebyshev map.

The expression of Chebyshev map is as follows:

(2) $$\begin{array}{c} z_{i+1}=\cos\left(w\left(\text{arc}\;\cos{\;z}_{i}\right)\right),\;-1\leq z_{i}\leq 1, \end{array}$$

where *w* is the degree of Chebyshev map. Its corresponding invariant density is as follows:

(3) $$\begin{array}{c} \rho \left(z\right)=\frac{1}{\left(\pi \sqrt{1-z^{2}}\right)}. \end{array}$$

**Figure 1:**
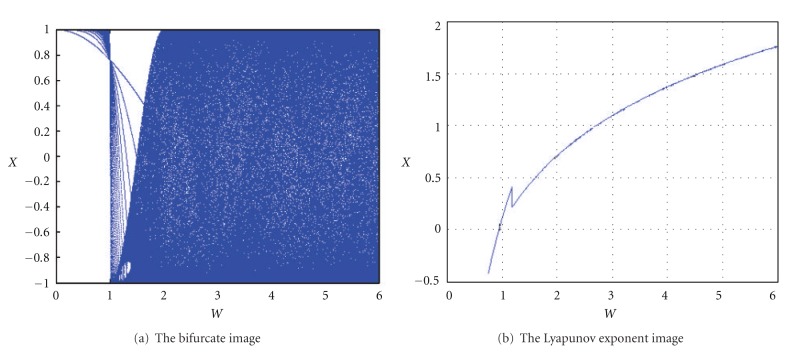
The bifurcate and Lyapunov exponent figures of Chebyshev map.

Chebyshev map has important properties of excellent cryptosystem [14, 15]. While *w* ≥ 2, the Lyapunov exponent of the Chebyshev map is positive, which predicates that Chebyshev map is chaotic, as shown in Figure 1.

Different sequences can be generated with different initial values; we get the sequence *S*_*n*_ ∈ {0,1} by

(4) $$\begin{array}{c} S_{n}=\{\begin{array}{cc} 0, & z_{n}\in \left[-1,0\right),\\ 1, & z_{n}\in \left[0,1\right]. \end{array} \end{array}$$

Finally, we can get the binary image *W*_*AX*_ by the XOR operation:

(5) $$\begin{array}{c} W_{AX}=W_{A}⊕S_{n}, \end{array}$$

which is a binary sequence to be embedded into the host image.

**Figure 2:**
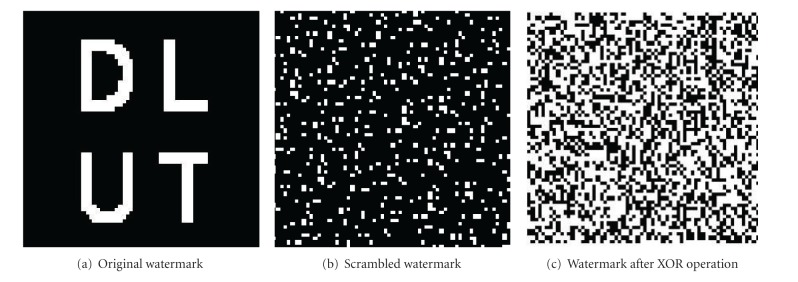
The preprocessing process of the original watermark.

 The preprocessing process, including the original image *W*, the scrambled image *W*_*A*_, and the image *W*_*AX*_, is shown in Figure 2.

### 2.2. Analysis of Wavelet Coefficients

**Figure 3:**
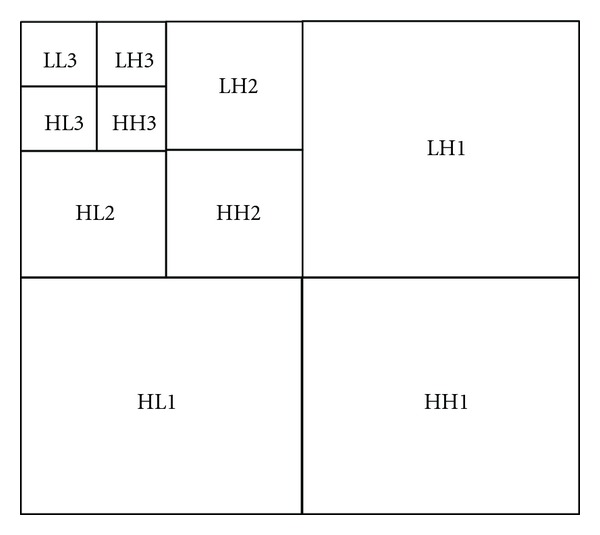
Wavelet subbands of 3-level.

The host image *I* of size *w* × *h* is transformed into wavelet coefficients using the *L*-level discrete wavelet transform (DWT). With *L*-level decomposition, we can get 3 × *l* + 1 subbands, as shown in Figure 3. The lowest band (approximation band) *LL*_*l*_ is the basic band of the decomposed wavelet, which includes most of the energy from the original image; it has a crucial effect on quality; therefore, *LL*_*l*_ frequency band is unsuitable to be modified.

Only embedding the watermark into *HH*_*l*_, *HH*_*l*−1_,…, *HH*_1_ subbands is also unsuitable, for they have the highest wavelet coefficients; the subbands can easily be eliminated and modified by lossy compression or other processing [14]. 

According to these characteristics, we design to adaptively adjusting, the high-frequency coefficient of the subbands *HL*_*l*_,  *LH*_*l*_, and *HH*_*l*_, to embed the watermark bits.

Generally, watermark embedding is realized by modifying some special values of pixels or transforming domain coefficients [16]. When the watermarked image receives some attacks, though the quality is still high, the pixel value or coefficient may be seriously eliminated, which will lead to the watermark detecting failed [17, 18]. Here we take advantage of the statistic characteristic, such as mean and variance of the *LH*_*l*_, *HL*_*l*_, and *HH*_*l*_ subbands, to modify the whole coefficients of them to dynamically embed the watermark and implement the compromise between quality and robustness.

### 2.3. Watermark Embedding

 After preprocessing the binary watermark image *W* to get *W*_*AX*_, we reshape *W*_*AX*_ to binary sequence *wm*; its length is still *n* × *n*. According to Human Visual System (HVS) [19], small changes to the blue component of color image are the most difficult to detect by human eyes; then we select the blue component to embed.

Here we improve the method in [9] to embed pre-processed watermark into the blue component of color image. Suppose the host color image is *I*. Firstly, we divide the blue component of *I* into a set of nonoverlapping *n* × *n* subblocks *b*_*j*_ ∈ *B*, *j* = 1,2, ..., *n* ^2^. Then a *l*-level DWT decomposition of each sub-block of image *I* is performed using Haar wavelet, and then compute the mean *E*_*j*_ of each wavelet block *S*_*D*_ by

(6) $$\begin{array}{c} E_{j}=\frac{1}{M}\left(\sum_{\left(x,y\right)\in S_{D}}c_{j}^{\left(l,s\right)}\left(x,y\right)\right), \end{array}$$

where *c*_*j*_^(*l*,*s*)^(*x*, *y*) is the wavelet coefficient of the *j*th sub-block, *l* is the level of wavelet decomposition, *s* refers to the three subbands of *LH*_*l*_,  *HL*_*l*_ and *HH*_*l*_. (*x*, *y*) is the coordinate for wavelet coefficient in the wavelet blocks, and *M* is the total number of wavelet coefficient in *S*_*D*_. 

For example, here we set *l* = 2, for the *j*th sub-block *b*(*j*) ∈ *B*; we use the *dwt*2() function in Matlab to perform a single-level two-dimensional wavelet decomposition. Finally, *c*_*j*_^(2,*s*)^(*x*, *y*) can be gotten by (9)

(7) $$\begin{array}{c} \left[LL_{j1},LH_{j1},HL_{j1},HH_{j1}\right]=dwt2\left(b\left(j\right){,}^{\prime }\text{haa}{\text{r}}^{\prime }\right), \end{array}$$

(8) $$\begin{array}{c} \left[LL_{j2},LH_{j2},HL_{j2},HH_{j2}\right]=dwt2\left(LL_{j1}{,}^{\prime }\text{haa}{\text{r}}^{\prime }\right), \end{array}$$

(9) $$\begin{array}{c} c_{j}^{\left(2,s\right)}\left(x,y\right)=\left[LH_{j2},HL_{j2},HH_{j2}\right]. \end{array}$$

The purpose of *E*_*j*_ is to keep the average of *c*_*j*_^(*l*,*s*)^(*x*, *y*) − *λE*_*j*_ in (10) and (11) close to zero.

If *wm*(*j*) = 1, we modify the whole wavelet coefficients in *S*_*D*_ by

(10) $$\begin{array}{c} {c_{j}^{\prime }}^{\left(l,s\right)}\left(x,y\right)=c_{j}^{\left(l,s\right)}\left(x,y\right)-\lambda E_{j}. \end{array}$$

If *wm*(*j*) = 0, we modify the whole wavelet coefficients in *S*_*D*_ by

(11) $$\begin{array}{c} {c_{j}^{\prime }}^{\left(l,s\right)}\left(x,y\right)=c_{j}^{\left(l,s\right)}\left(x,y\right)-\lambda E_{j}+P. \end{array}$$

Here *c*_*j*_′^(*l*,*s*)^(*x*, *y*) is the modified wavelet coefficient; the distribution of wavelet coefficient is modulated by *wm*(*j*). *s* refers to the three detail subbands of *LH*_*l*_, *HL*_*l*_, and *HH*_*l*_. That is to say, embedding “0” means that the coefficient mean of wavelet block *S*_*D*_ is *P*, and embedding “1” means 0. *λ* refers to the magnification of *E*_*j*_; here we set *λ* = 1.5 to ensure the range of threshold *Th* larger enough to extract the watermark.

Because the variance is bigger in the textures and edges than that in the smooth region, we use the variance of the coefficient as the modulation coefficient *P* to control the dynamically embedding intensity [19]. Raising embedding intensity within the definition can enhance the robustness [15]. So *P* can be defined by

(12) $$\begin{array}{c} P=A+T\left(j\right). \end{array}$$

*T*(*j*) is defined as

(13) $$\begin{array}{c} T\left(j\right)={\left(\sqrt{{\left(\sum_{\left(x,y\right)\in S_{D}}\left(c_{j}^{\left(l,a\right)}\left(x,y\right)-c_{j}^{\left(l,b\right)}\left(x,y\right)\right)-\lambda E_{j}\right)}^{2}}\right)}^{\beta }. \end{array}$$

We can find that *T*(*j*) is the local variance of the *j*th wavelet sub-block except the approximate sub-band *LL*_*l*_, and *β* is a constant. In (12), *A* is the intensity coefficient, which ensures to embed some watermark in the smooth region while *T*(*j*) is approximately 0. So (11) can be fully expressed by (14).

(14) cj′(l,s)(x,y)=cj(l,s)(x,y)−λEj+A+((∑(x,y)∈SD(cj(l,a)(x,y)−cj(l,b)(x,y))−λEj)2)β.

Using (10) and (14) we embed watermark by modify the wavelet coefficients. Finally, the IDWT is applied to each block, to get the watermarked image *I*_*w*_.

### 2.4. Watermark Extraction

The watermark can be extracted correctly from the watermarked image, without the original host image or the watermark image, so the scheme belongs to blind watermark algorithm. The transformed coefficients are compared with the threshold Th of the coefficients, those coefficients above the threshold are retained, and all the others are discarded [20].

The extracting process is as follows, which is mirroring the embedding process.

**Step 1**

We divide the blue component of the watermarked color image *I* into a set of nonoverlapping *n* × *n* subblocks.

**Step**

To extract the embedded bit from a sub-block, the *L*-level DWT decomposition of each block is firstly obtained. Then we compute Sum(*j*), the coefficients sum of the wavelet block *S*_*D*_ using

(15) $$\begin{array}{c} \text{Sum}\left(j\right)=\sum_{\left(x,y\right)\in S_{D}}{c^{\prime }}_{j}^{\left(l,s\right)}\left(x,y\right). \end{array}$$

For all the wavelet blocks of *j* = 1,2, ..., *n* ^2^,

(16) $$\begin{array}{c} \text{Sum}\left(j\right)\in \left[r1,r2\right],\;\text{if}\;S_{D}\;\;\text{carriesbit}\;“1",\\ \text{Sum}\left(j\right)\in \left[r3,r4\right],\;\text{if}\;S_{D}\;\;\text{carriesbit}\;“0", \end{array}$$

where *r*1 < *r*2 < *r*3 < *r*4, then we can get the range of Th ∈ (*r*2, *r*3).

**Step 3**

Sum(*j*) is compared with the threshold Th, to decide whether the coefficient carries a bit “1” or “0” using

(17) $$\begin{array}{c} wm\left(j\right)=\{\begin{array}{cc} 1 & \text{Sum}\left(j\right)<\text{Th},\\ 0 & \text{Sum}\left(j\right)\geq \text{Th}, \end{array} \end{array}$$

where *wm*(*j*) is the extracted bit.

**Step**

Finally, the extracted watermark bits are recovered into a binary image according to the inverse process.

During the embedding and extracting processes, the iteration times *t* of Arnold transform, the initial value of Chebyshev map, the size *n* × *n* of each block, the values of *A*, *λ*, and *β*, the threshold Th, and the type of wavelet are all served as keys, which can guarantee the security of the watermarked image; it is impossible to extract the watermark without these keys.

## 3. Experimental Result Analysis

### 3.1. Get the Effective Range of the Threshold in Experiment

 The threshold is a critical factor, which can ensure to extract the watermark correctly from watermarked image under some attacks, and some papers only provide a fixed value [3, 7, 8]. The proposed scheme provides an exact range of the threshold by testing dozens of images, and it keeps constant even if the watermarked image has been attacked in some degree.

**Figure 4:**
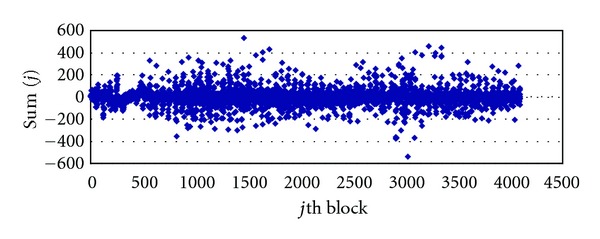
Distribution: Sum(*j*) of the wavelet blocks *S*_*D*_ before embedding.

Figure 4 is Image1 distribution of Sum(*j*) before embedding; we can find that most of the coefficients in the detail subbands after DWT are close to zero, and the mean of each detail sub-band is approximately zero. 

**Figure 5:**
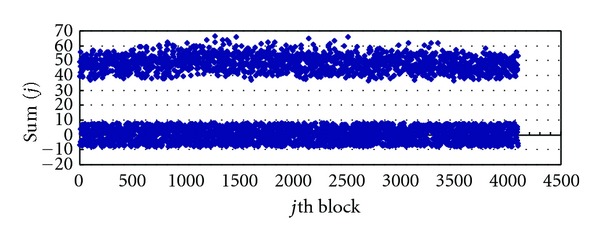
Distribution: Sum(*j*) of *S*_*D*_ after embedding (*β* = 0.218).

**Figure 6:**
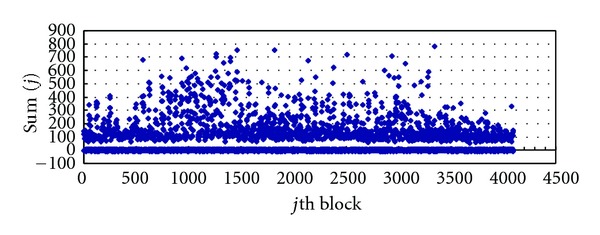
Distribution: Sum(*j*) of *S*_*D*_ after embedding (*β* = 0.818).

Figures 5 and 6 are the Image1 distribution of Sum(*j*) after embedding with *β* = 0.218 and *β* = 0.818; from them we can find that when *β* = 0.218, the distribution of Sum(*j*) is relatively concentrated, but when *β* = 0.818, the values of Sum(*j*) distribute in larger range. The distribution of other images, such as Image3 and Airplane, is similar to Image1 when we set *β* = 0.218. 

Experiment results demonstrate that when we set *β* ∈ [0.218,0.818], the threshold Th ∈ (10,35), which is consistent with the result of (16), the range of Th  watermark can ensure the watermark to be extracted clearly from the watermarked images.

### 3.2. The Relationship between *β* and PSNR

**Figure 7:**
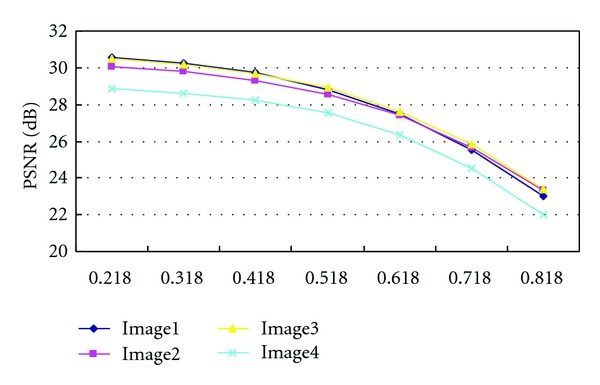
The relationship between *β* and PSNR (dB).

**Figure 8:**
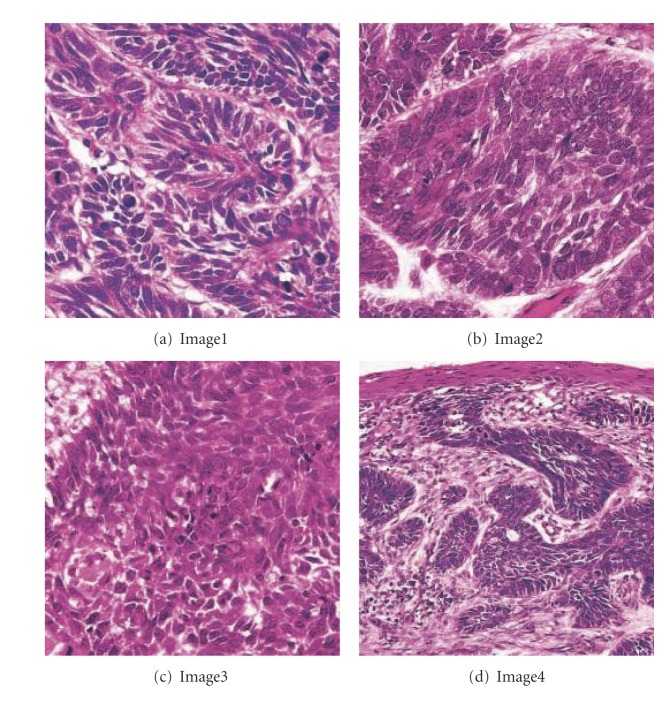
Sample images.

The quality of watermarked image is based on the PSNR [12]. From Figure 7 we can find that the PSNRs decrease smoothly with the increase of *β* ∈ [0.218,0.818], which can increase the embedding intensity *P*. What's more, Figure 7 shows that the PSNR values of Image1, Image2, and Image3 are similar, and they are greater than that of Image1, for they have richer edges and borders than Image4, as shown in Figure 8.

## 4. Experimental Results and Key Space

### 4.1. Experimental Result

**Figure 9:**
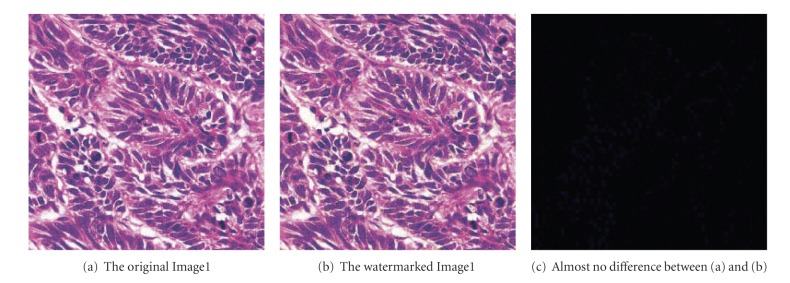
The original and the watermarked Image1, and the difference between them.

Here we made a 64 × 64 binary image as the watermark. Some standard color images with the size of 512 × 512 × 24 bits, including Image1, Airplane, and Image3, are being tested. The size of the sub-block is set to 8 × 8, *λ* = 1.5, *l* = 2, when *A* ∈ [4,22], *β* ∈ [0.218,0.818], and Th ∈ (10,35); the results of watermarked images are satisfactory, as shown in Figure 9.

### 4.2. Key Space Analysis

 The high sensitivity to initial conditions is inherent to any chaotic system. To provide an secure algorithm, the key space should be large enough to make any brute force attack ineffective. Our scheme has some of the following secret keys: (1) for the Arnold cat map, the initial values of *x*_0_ and  *y*_0_, the iteration times *t*; (2) Chebyshev maps, the initial value *z*_0_, and the parameter *w*.

For the Arnold cat map, *x*_0_, *y*_0_ ∈ [1, *n*] and  *t* ∈ [1,256], here *n* = 64. For the Chebyshev maps, when the tiny change in the initial value Δ*z*_0_ = 10^−16^, the scrambled watermark image is completely different. A large number of experimental results indicate that the key spaces for initial values are *S*_*z*_0__ = 10^16^. Similarly, the variation of the parameter *w* in the chaotic region is between 2 and 6 with a step of 10^−16^, so *S*_*w*_ ≈ 4 × 10^16^.

Finally, the total key space is *S* = *S*_*x*_0__ × *S*_*y*_0__ × *S*_*t*_ × *S*_*z*_0__ × *S*_*w*_ ≈ 4.19 × 10^38^, which is larger than 2^100^, so even the scrambled watermark is extracted; it's difficult to recover it.

## 5. Attack Test Results

The attacks to the whole watermarked image can modify the red, green, and blue components simultaneously. The modifications in the red or green component are easier to be observed, that is only because of the Human Visual System. There is no evidence to show that the modification in the blue component can be more robust than the other components.

The peak signal-to-noise ratio (PSNR) is used to evaluate the quality between an attacked image and the original image. For the sake of completeness, we list the PSNR formula as follows:

(18) PSNR=10×log10255×2551/(Ih×Iw)∑x=0Ih−1∑y=0Iw−1[f(x,y)−g(x,y)]2dB,

where *I*_*h*_ and *I*_*w*_ are the height and width of the image, respectively. *f*(*x*, *y*) and *g*(*x*, *y*) are the values located at coordinates (*x*, *y*) of the original image and the attacked image, respectively.

 After extracting the watermark, the normalized correlation coefficient (NC) is computed using the original watermark and the extracted watermark to judge the existence of the watermark and to measure the correctness of an extracted watermark. It can be defined as

(19) $$\begin{array}{c} \text{NC}=\frac{1}{W_{h}\times W_{w}}\sum_{i=0}^{W_{h}-1}\sum_{j=0}^{W_{w}-1}W\left(i,j\right)\times W^{\prime }\left(i,j\right), \end{array}$$

where *W*_*h*_ and *W*_*w*_ are the height and width of the watermark, respectively. *W*(*i*, *j*) and *W*′(*i*, *j*) are the watermark bits located at coordinates (*i*, *j*) of the original watermark and the extracted watermark.

### 5.1. Attack of Noise

 Noise attack is a common attack during the transmission of the watermarked image on the network [21]. In the experiment, we add salt and pepper noise and Gaussian white noise to Image1 image, as shown in Table 1.

For the watermarked image with salt and pepper noise, with the increase of noise density, more and more noise points appear in the extracted watermark, when the density is set to 0.05, the NC is 0.5309, and the extracted watermark can still be distinguishable. Similarly, for the watermarked image attacked by Gaussian white noise, when the mean value is set to zero, more noise points appear in the extracted watermark with the increase of variance; when variance is set to 0.01, the extracted watermark can still be distinguishable.

### 5.2. Attack of JPEG Compression

 JPEG lossy compression is the most common image compression technique. The watermarked image Image1 is compressed using JPEG with different value of *β*, horizontal resampling by 2 : 1, and compression ratios, 0.80, 0.65, 0.50, 0.25, and 0.10 respectively. The results obtained are summarized in Table 2; the distinguishable watermark can still be extracted even the ratio is set to 0.10, so the algorithm is very robust against compression.

### 5.3. Attack of Cropping

The algorithm is also very robust against the cropping operation. In order to resist true cropping, that is, only a portion of the image is kept and the remaining part is thrown away, the width and height of the host image, along with the other keys, are sent to the detector. If the detector receives a portion of the image, he can recover it to the original size by zero padding, to extract the watermark.

 After randomly selecting the position and crop 5%, 10%, 25%, 50%, and 75% of the watermarked image, respectively, then extract the watermark, as shown in Table 3. When 50% of the whole image is cropped, we can still extract the distinguishable watermark. That is to say, the algorithm has high robustness against cropping.

After cropping the randomly selected position, and filling the cropped region with zero, the extracted watermark before and after the reverse transformation is shown in Table 4; from it we can find that the randomly cropping has no effect on the extracted watermark.

Without the preprocessing to scramble the watermark, the four letters “DLUT” on the original watermark may be unexpectedly cropped; that is, the extracted watermark may be fragmentary, so scrambling the watermark by Arnold cat map in Section 2.1 can help to make the bits randomly distributed, which contributes to the robustness.

### 5.4. Attack of Brightness

**Figure 10:**
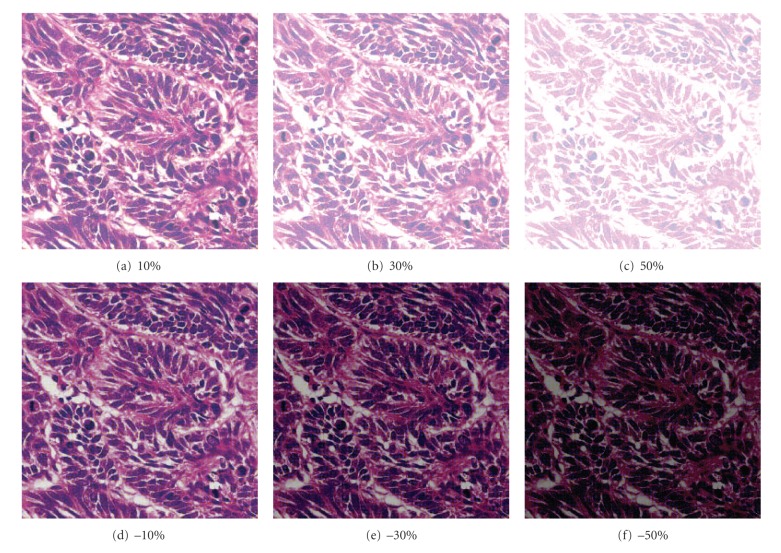
Image1: enhance and decrease the brightness.

 We increase and decrease the brightness by 10%, 30%, and 50%, respectively, as shown in Figure 10. The extracted watermarks demonstrate that increasing the brightness has less effect on the watermark than decreasing the brightness, as shown in Table 5. So the algorithm has high robustness against the attack of brightness.

### 5.5. Comparison

Finally, we use Image1 with *β* = 0.218 to compare our algorithm with the algorithms proposed in [4, 11, 18]. The results show that our algorithm gets advantages over the others in the attacks of median filter, compression, rotation, and cropping, as shown in Table 6.

## 6. Conclusion

 We propose a dynamic block-based blind watermark algorithm based on a 2-level DWT, using Haar filter to embed a preprocessing binary image into the blue component of the color pathological image. By analyzing the coefficients' characteristic after wavelet decomposition, we select the detail subbands to embed watermark. During the embedding procedure, the statistic characteristics of the mean and variance are applied to dynamically adjust the embedding intensity, so as to generate the effective range of threshold to extract the watermark. Experiment results demonstrate that the quality of the watermarked image, which is based on the PSNR, decreases smoothly with the increase of the embedding intensity, and the algorithm is robust against common distortions, especially, getting advantages over noise, compression, cropping, rotation, and brightening.

## Figures and Tables

**Table 1:** Noise attacking results for Image1.

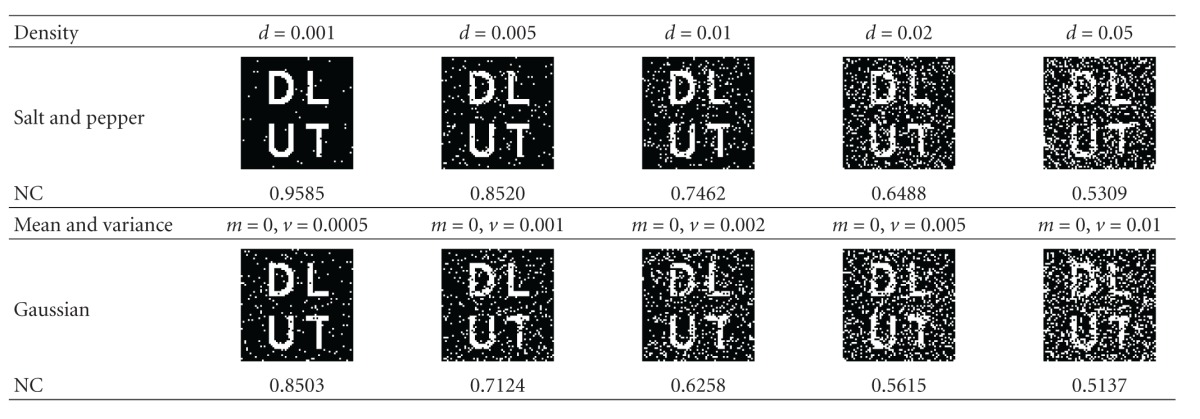

**Table 2:** Compression results with different ratios.

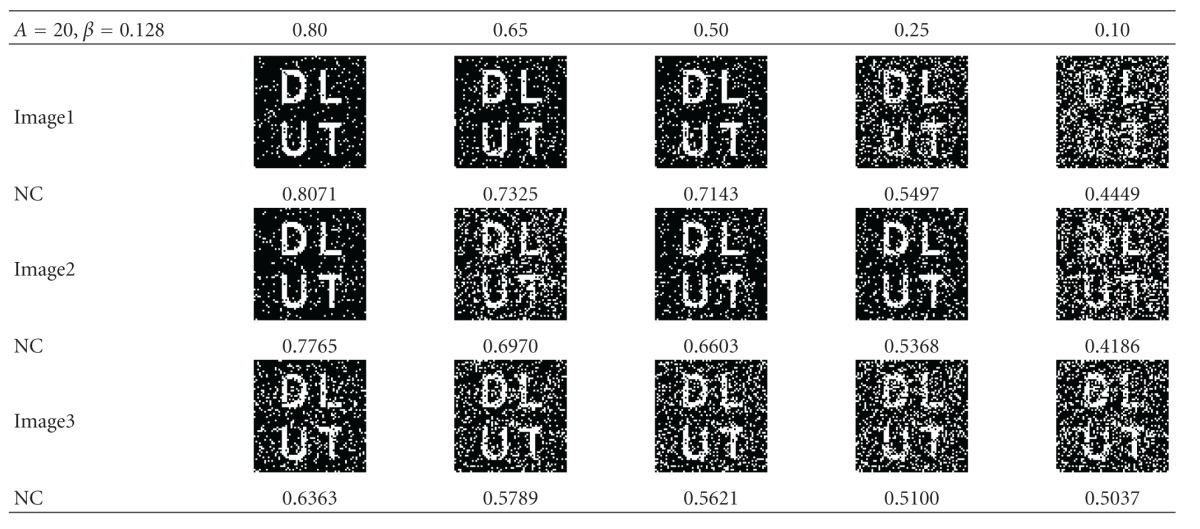

**Table 3:** Extracting results for Image1 and Image2 after being cropped.

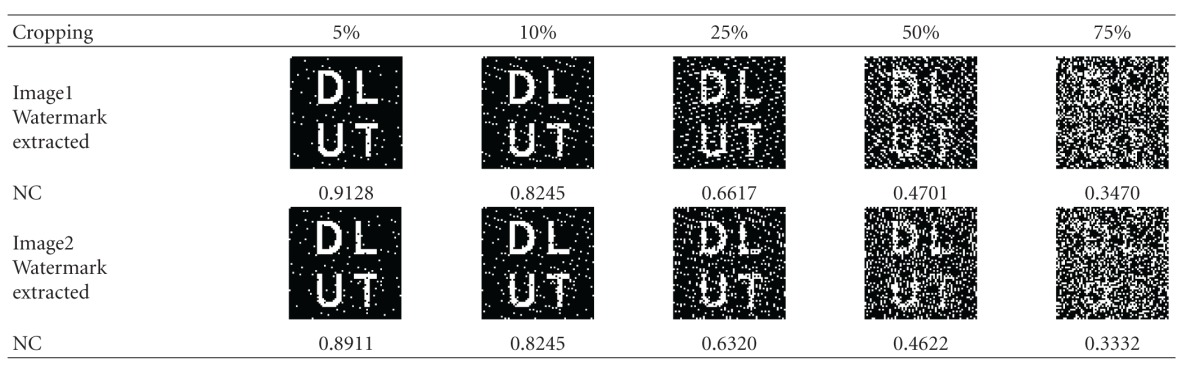

**Table 4:** Randomly select the cropping position and the extracting result.

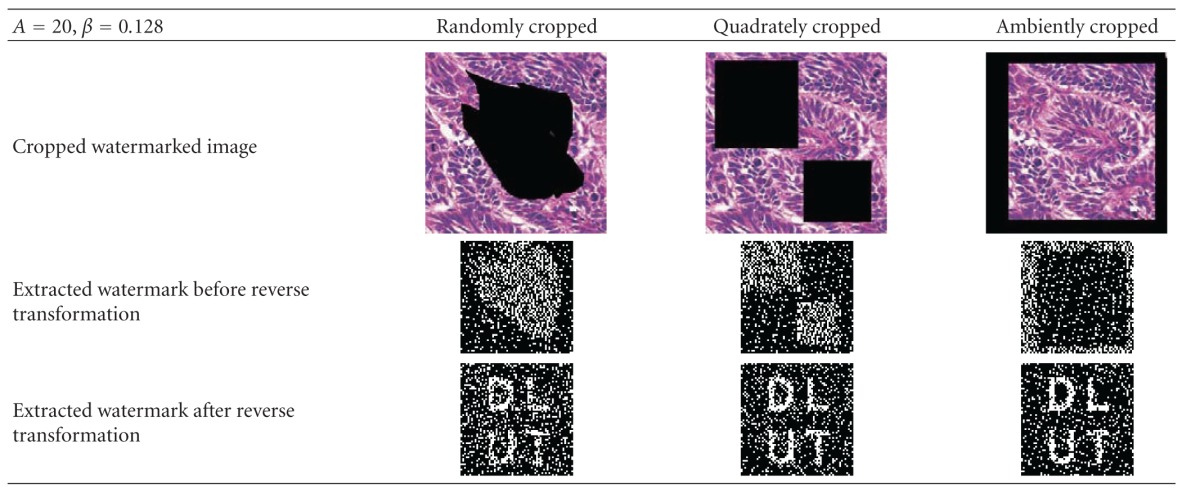

**Table 5:** Extracting results from the image with enhanced and decreased brightness.

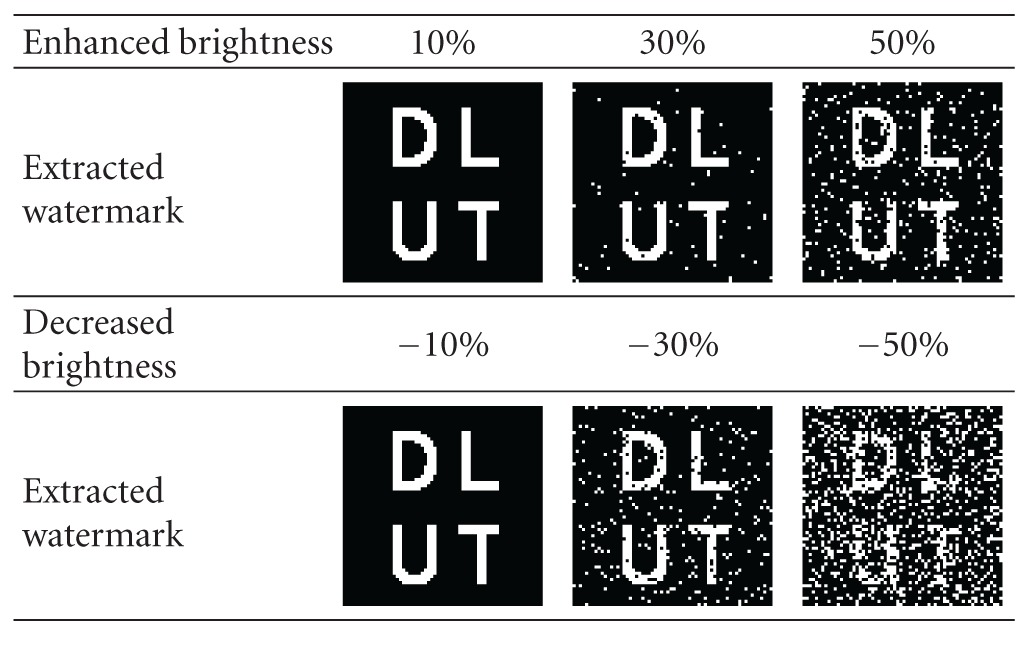

**Table 6:** Compare the proposed method with the algorithms in [4, 11, 18].

Attacks	NC			
	Reference [4]	Reference [11]	Reference [18]	Our algorithm
	(PSNR = 42.02 dB)	(PSNR = 44.73 dB)	(PSNR = 42.98 dB)	(PSNR = 31.14 dB)
Median filter (3 × 3)	0.90	0.88	0.93	1
JPEG (ratio = 0.80)	0.99	NA	1	0.9997
JPEG (ratio = 0.50)	0.96	0.95	0.96	0.9976
JPEG (ratio = 0.10)	0.34	0.33	0.32	0.9987
Rotation (degree: 0.25°)	0.59	0.61	0.65	0.9897
Rotation (degree: −0.25°)	0.60	0.65	0.72	0.9900
Cropping 1/4	0.66	0.60	0.67	0.8561
Scaling 256 × 256	0.88	0.71	0.85	0.9999
